# Correlates of Balance and Aerobic Indices in Lower-Limb Prostheses Users on Arm Crank Exercise

**DOI:** 10.3390/s21206917

**Published:** 2021-10-19

**Authors:** Gary Guerra, John D. Smith

**Affiliations:** 1 Sirindhorn School of Prosthetics and Orthotics, Faculty of Medicine, Siriraj Hospital, Mahidol University, Bangkok 10700, Thailand; gary.gue@mahidol.edu; 2 Department of Counseling, Health & Kinesiology, Texas A&M University-San Antonio, San Antonio, TX 78244, USA

**Keywords:** amputee, aerobic capacity, arm crank exercise, center of pressure, prosthetics, balance

## Abstract

Background: The HUMAC Balance System (HBS) offers valid measurement of balance, and the arm crank exercise test (ACE) is a valid measure of physiological capacity. Neither have been used to evaluate associations between balance and physiological capacity in lower-limb amputees. Methods: Thirty-five participants with lower-limb amputations were recruited. Standing balance (center of pressure) was evaluated during eyes opened (EO) and eyes closed (EC) conditions using the HBS. Participants performed ACE graded exercise testing (GXT) to evaluate aerobic capacity. Spearman’s *rho* was used to identify relationships between variables. Cut-points for three groups were generated for time on ACE. Mann–Whitney *U* tests were used to explore significant differences in variables of balance and ACE between low and high performers. Results: Relationships between variables of eyes open displacement (EOD), eyes open velocity (EOV), eyes closed displacement (ECD), and eyes closed velocity (ECV) were significant (*p* < 0.05), and high performers with EO also performed best with EC. Longer exercise times were significantly associated with increased HR_peak_, VO_2peak_, VE_peak_, and RER_peak_ (*p* < 0.05). HR_peak_ (143.0 ± 30.6 b/min), VO_2peak_ (22.7 ± 7.9 and 10.6 ± 4.7 mL/kg/min), VE_peak_ (80.2 ± 22.2 and 33.2 ± 12.7 L/min), and RER_peak_ (1.26 ± 0.08 and 1.13 ± 0.11) were significantly greater in high performers than low performers, respectively (*p* < 0.05). There was no significant association among VO_2peak_ and any balance task variables; however, there were significant associations between some balance and physiological variables. Conclusions: Findings differentiated high and low performers; however, participants were still well below able-bodied norms of physical capacity. Training to mitigate deconditioning is suggested.

## 1. Introduction

An essential requisite for prosthesis user mobility is stable upright balance, and studying standing balance aides clinical decision making [1]. Measurement of standing balance involves analyzing the center of pressure (COP), which influences the center of mass by way of ankle and hip torques in the sagittal and coronal planes, respectively [2]. Limb amputation increases the demand for postural control due to increased asymmetrical loading on the non-amputated limb [3,4], and it increases use of the ankle strategy and somatosensory input of the lower limbs [5,6]. Research has evidenced greater COP displacements in those with lower-limb amputations (LLA) compared to able-bodied persons [6], identified greater levels of COP displacement at higher amputation levels [7], and observed that shorter residuum lengths increase sway area and velocity [8].

Balance measurements are traditionally performed with expensive platforms that are restricted to analysis within motion analysis laboratories. However, current technologies such as the Wii balance board (Wii, Nintendo, Japan) have offered portable, affordable, and reliable postural sway measurements [9,10,11]. The HUMAC Balance System (HBS) (CSMi Inc, Stoughton, MA, USA) is mechanically based on the Wii Balance Board and, like the ACE, offers small footprint portable data collection. Although it is surprising that there is no published research evaluating balance using the HUMAC Balance system in amputees, the use of such portable devices would allow easy assessment in persons wearing a prosthesis.

In a similar vein, and equally as important to prosthetic rehabilitation, is physiological capacity. It is well known that energy expenditure of walking for prosthesis wearers is greater than that for able-bodied persons [12,13], greater in dysvascular amputees [14], and even greater for those with more proximal amputations [15]. A comprehensive diagnostic evaluation of capacity can be determined from administering a graded exercise test (GXT) with a metabolic analyzer to evaluate cardiovascular and cardiopulmonary functioning. The test is a criterion for determining cardiorespiratory fitness and maximal oxygen consumption (VO_2_max) [16]. Treadmills, cycle ergometers, and arm crank ergometers have all been utilized for GXT, and cycle ergometers and even arm crank ergometers (ACEs) have been employed in aerobic capacity studies with prosthesis-wearing participants [17,18]. In addition to measurement of VO_2_max, ACE provides measures of workload (watts) and time to exhaustion (TTE). Arm crank exercise offers similar capabilities of other ergometers such as the ability to determine VO_2_max, measures of workload (watts), and time to exhaustion (TTE). However, the ACE is portable, whereas treadmill and cycle ergometers are often confined to a laboratory. Furthermore, in ACE the subjects remain seated during testing, thereby removing possible effects the prosthesis may have on the test.

There is evidence that residuum length [19], cause of amputation [20], and even reduced somatosensory status [21] can each influence amputee balance. However, aerobic capacity, which is a clinical vital sign for health [22], and its possible association with standing balance of lower limb amputees is not precisely clear. Furthermore, very few studies have employed the ACE to evaluate cardiorespiratory fitness performance in LLA. These studies have focused on amputee responses to combined arm–leg ergometry [18], or have been submaximal in testing protocol [23]. As of yet, a portable and clinically implementable performance battery for evaluating prosthesis user balance and physiological capacity has not been established. Within this context, the specific aim of this work was to utilize the HBS to evaluate balance and examine the association between lower-limb amputee balance and aerobic capacity on ACE.

## 2. Materials and Methods

This study was approved by the Texas A&M University San Antonio Institutional Review Board (Log#2017-37), and all participants signed an informed consent before participating. Thirty-five individuals with lower-limb amputations and without major limb pathology or underlying conditions that would have influenced standing balance or ACE participated in the study (Table 1). Participants completed a Physical Activity Readiness Questionnaire (PAR-Q) to assess cardiovascular risk that would exclude them from participating [24,25]. Body mass and height were assessed while wearing prostheses and shoes using a digital weight scale (Detecto, SlimPRO, Webb City, MO, USA) and a stadiometer (Seca 213, Hamburg, Germany), respectively.

### 2.1. Balance

Participants first performed two separate standing static balance tasks using the HUMAC Balance System, which is an electronic balance board interfaced with the HUMAC software on a laptop. Calibration was performed following manufacturer guidelines prior to balance testing. Participants mounted the HBS and were asked to stand in their normal standing position with hands by their sides. Assisted devices (canes, crutches, etc.) were not allowed, and an investigator was situated to the side and behind participants in case balance was lost. A 5 cm × 5 cm piece of colored paper taped to the wall at the participant’s eye level served as the sight target. Prior to testing, participants stood quietly in front of the platform for one minute, after which they mounted the scale for testing. Participants performed two 30 s static balance tasks in the following order: double-limb support standing with eyes open (EO) and eyes closed (EC). Each of the system’s four linear force sensors (strain gauges) on the corners of the HBS were sampled at ~100 Hz [26,27]. Data from the HBS were filtered and analyzed using the system software. Center of pressure displacements during each task were collected to derive metrics of mean center of pressure displacement (cm) with eyes open (EOD) and eyes closed (ECD), center of pressure displacement velocity (cm/s) with eyes open (EOV) and eyes closed (ECV), and stability score with eyes open (EOS) and eyes closed (ECS). The software calculated the stability score as the percent of the patient’s tilt relative to 6.25 degrees, which is the limit set in the manufacture’s software. This system has been shown to be a valid instrument in static conditions [28].

### 2.2. Arm Crank Ergometer (ACE)

**Figure 1:**
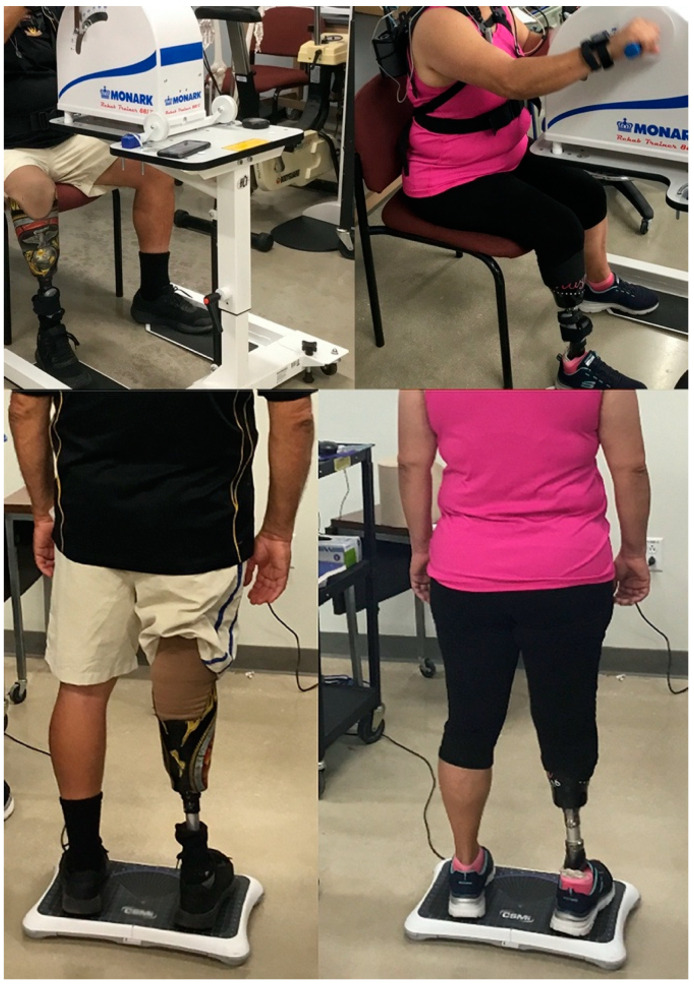
Example setup for the balance and arm crank exercise assessment.

Participants were fitted with a COSMED K5 portable metabolic analyzer (Cosmed, Rome, Italy) interfaced with a Polar FT1 chest strap heart rate monitor (Polar, Kempele, Finland). A Monark 881E (Monark, Varberg, Sweden) secured to a HealthCare International upper body exercise table (Langley, WA, USA) was used to administer ACE. Participants were provided as much time as needed to crank with no resistance for familiarization, during which time the ergometer was adjusted for comfort. Test cadence was 60 revolutions per minute (RPM), which was viewable on a digital monitor interfaced with the ergometer. The ACE consisted of two-minute stages with an initial resistance of 16 W and increased 16 W every stage until exhaustion. If a participant reached 100 W, which is the maximal watt level on the Monark 881E, then that resistance was maintained and RPM increased by 5 W every stage. The test was terminated when participants failed to maintain the required RPMs or when they felt they could no longer continue and voluntarily stopped. The K5 was marked for oxygen consumption (VO_2_peak), ventilation (VE), respiratory exchange ratio (RER), and heart rate data at the end of every stage. At the end of exercise, a rating of perceived exertion scale (RPE, Borg’s 6–20) was shown to participants, time to exhaustion (TTE) was recorded, and participants were then given a 2 min cool-down where they cranked at 60 RPMs with no resistance (Figure 1).

### 2.3. Statistical Analysis

All data were analyzed with IBM SPSS v25 (Chicago, IL, USA). Normality of data was calculated by dividing the skewness statistic and the kurtosis statistic with their respective standard errors and comparing with ±1.96. If these statistics were outside ± 1.96 then the curve was not normally distributed. Since much of the data were found to be outside the acceptable limits for normality (Table 2), non-parametric tests were used to analyze results. Spearman’s *rho* was used to identify relationships between variables. Cut-points for three groups were generated for time on ACE, with the highest group cut-point at 470 s and above, the middle group at 356–469 s, and the lowest performing group at 355 s and below. Three groups were created, and only the highest (*n* = 11) and lowest (*n* = 12) groups were used for comparison in order to clearly distinguish higher and lower performers.

Mann–Whitney *U* tests were used to explore for significant differences in balance task and ACE variables between the group with the shortest times (lowest performing group) and the group with the longest times (highest performing group). Alpha was set at 0.05 for all tests.

## 3. Results

**Figure 2:**
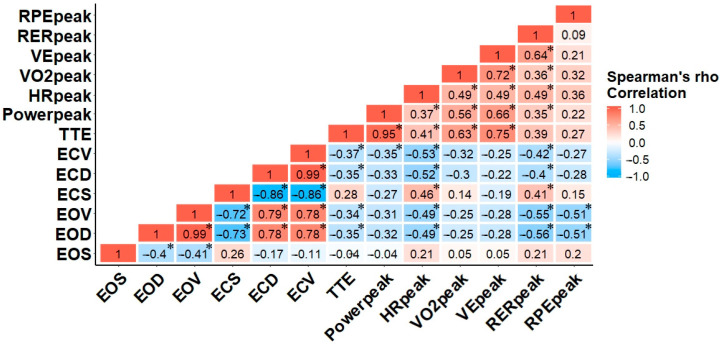
Correlation matrix for Spearman’s *rho* of balance task and arm crank exercise variables. The colors of the scale bar denote the direction of the correlation with 1 indicating a perfect positive correlation (dark red) and −1 indicating a perfect negative correlation (dark blue) between balance and physiological variables. * correlation is significant at the (0.05) level (2-tailed).

Figure 2 illustrates the magnitude of Spearman’s *rho* for all variables. Within the balance task, the relationships among the rankings of EOD, EOV, ECD, and ECV were significant (*p* < 0.05), suggesting those who performed best with eyes open also performed best with eyes closed. While longer exercise times were significantly associated with increased heart rate and cardiorespiratory variables, only HR_peak_, VO_2peak_, VE_peak_, and RER_peak_ were significantly correlated with each other, respectively (*p* < 0.05). Although there was no significant association among VO_2peak_ and any balance task variables, HR_peak_ was moderately and significantly associated with balance task in both EO and EC conditions. Interestingly, RER_peak_ was the only cardiorespiratory variable related to the EO and EC balance tasks. Finally, the only significant relationships for RPE existed in the EO balance task, *p* < 0.05 (Figure 2) [29].

Mann–Whitney *U* tests indicated the only significant difference in mean rank balance task scores between highest performers (HP) and lowest performers (LP) on ACE were in EOD and ECV, *p* < 0.05. There were no other significant differences in any of the balance scores between highest and lowest performers. There was a significant difference between HP and LP for TTE (612 ± 105 and 270 ± 63 s, respectively), Power_peak_ (83.2 ± 14.4 and 40.0 ± 8.3 W, respectively), VO_2peak_ (22.7 ± 7.9 and 10.6 ± 4.7 mL/kg/min, respectively), VE_peak`_ (80.2 ± 22.2 and 33.2 ± 12.7 L/min, respectively), and RER_peak_ (1.26 ± 0.08 and 1.13 ± 0.11, respectively), *p* < 0.05. Interestingly, there was no significant difference in HR_peak_ between highest (150.4 ± 23.5 b/min) and lowest (136.8 ± 34.8 b/min) performers (Table 3).

## 4. Discussion

Results of this study differentiated high and low performers, as those with better indices of balance with eyes open also had better balance with eyes closed. The HP group displayed better performance on balance metrics when compared to the LP group and performed significantly better in EOD and ECV. Those performing longest on ACE were more likely to have greater cardiorespiratory and heart rate responses than those who could not achieve greater work rates. The results, herein, are in agreeance with prior research using force plates that demonstrated comparable results in amputees of 46.8 ± 16.6 cm [30] and 31.6 ± 12.9 cm [31] during EOD tasks. Two earlier investigations in able-bodied individuals performing EO balance tasks have reported smaller COP displacements of 38.7 ± 6.7 cm [10] and 36.03 ± 7.88 cm [32]. These small but noticeable differences between able-bodied and LLA populations grow during eyes closed tasks. As an example, previous scholarship observed marked differences in COP excursions during soft-surface eyes closed tasks, 117.4 ± 61.9 cm and 90.4 ± 56.0 cm for amputee and able-bodied groups, respectively [31]. In the current study, the ECD balance task elicited over a two-fold increase (45.2 ± 39.4 cm) over the EOD condition.

Center of pressure velocity followed COP displacement trends, with greater velocity observed during eyes closed tasks. These data are consistent with a previous study that observed a velocity of 1.25 cm/s in transtibial and transfemoral prosthesis users [33]. Our EO velocity condition values, whilst fairly similar to those of Park et al. (2014) [32], were still greater in the EC condition. Interestingly, these authors instructed their participants to cross arms across the midline during balance testing. Although this might appear to be a minor detail, previous studies have observed improvement in clinical balance tests when arms were not restricted compared to when arms were restricted [34].

It is clear that assessment of balance in prosthesis wearers is advantageous for mobility purposes, but it is also important to evaluate aerobic fitness in this population as well. Past studies using ACE have elicited VO2_peaks_ of 25.9 ± 1.6 mL/kg/min in younger healthy untrained men [35] and 24.9 ± 4.0 mL/kg/min in younger healthy women [36]. A larger study with healthy men and women showed similar results, such as peak values for VO_2_ (26.9 ± 6.8 mL/kg/min), VE (71.6 ± 21.3 L/min), RER (1.16 ± 0.10), RPE (18.9 ± 1.10), and power (97.0 ± 31 W), thus demonstrating the usefulness of ACE to determine cardiorespiratory fitness levels [37]. Lower VO2peak (16.4 ± 4.1 mL/kg/min), VEpeak (51.5 ± 13.8 L/min), and peak power (78.7 ± 23.9 W) are consistent with increases in age [38], and these values are comparable with the current study (Table 2). Moreover, our measured VO_2peak_ values are lower than those seen in able-bodied [37], but are as expected for amputees [39,40].

In the current study, peak heart rates between LP (125 ± 29 b/min) and HP (151 ± 29 b/min) were not significantly different, although trends were elevated in the latter group. Peak heart rates were correlated with TTE and VO_2peak_. While this is unexplained because of the direct linear relationship between workload, VO_2_, and heart rate, it may be the effect of medications that were not disclosed by some participants. As noted in past literature, much of LLA exhibit poor fitness, but with training this fitness can increase [39], and the current study demonstrates the usefulness of ACE to identify fitness in this population.

It is plausible to surmise that the more fit an individual, the better balance that individual will have. Moreover, balance and aerobic capacity, both of which are deteriorated post amputation [41], can improve after rehabilitation and endurance training [40,41,42,43]. Interestingly this study found that fitness levels measured by ACE were not correlated with balance. The second aim of this paper was to explore associations between balance and aerobic capacity. This is the first study to explore this relationship in LLA via ACE testing. Participants in the HP group who achieved longer TTE did not necessarily have better balance performance indicators, and moreover, performing well on the ACE did not directly translate to better balance ability. While TTE showed a moderate-weak relationship with EOD, EOV, and ECD, none of the cardiorespiratory variables presented a consistent finding to firmly establish this. This may be due to LLA prioritizing upper body activities as opposed to lower body activities, thereby compromising balance performance.

### Limitations

A number of factors may serve as limitations in the current study. First, a smaller sample and middle age range may limit broader generalization of these findings. Second, as per agreement of the study, participants were not required to divulge their amputation etiology. Moreover, a history of wheelchair use could also affect participant responses on ACE. Future research should be conducted to recruit a larger sample of participants with specific amputation etiologies to elucidate possible differences in balance and aerobic capacities between these groups. Differences between participants with different amputation etiologies have been reported before [44]. In addition, walking ability was not evaluated, which can provide important additional information on participant functional ability. Finally, non-disclosure of medications, such as blood pressure medications, might have influenced heart rate responses and hindered participant ability to achieve a peak heart rate.

## 5. Conclusions

The HBS and ACE successfully permitted the evaluation of balance and aerobic capacity in the lower-limb prosthesis wearer. These data differentiated high and low performers and elucidated distinct associations between balance and physiological variables. These instruments provide a means for evaluating and differentiating both cardiorespiratory fitness and balance indices in LLA. Further research must be performed to help establish whether these instruments can be useful to practitioners in clinical settings.

## Figures and Tables

**Table 1:** Participant characteristics.

	Total (*n* = 35)	Male (*n* = 19)	Female (*n* = 16)
Age (y)	48.5 ± 14.8	50.2 ± 14.6	46.5 ± 15.1
Height (cm)	171.0 ± 8.5	176.5 ± 5.6	164.4 ± 6.2
Mass (kg)	86.0 ± 24.6	99.1 ± 21.8	70.5 ± 18.0
BMI (kg/m^2^)	29.2 ± 7.2	31.8 ± 6.9	26.1 ± 6.2
Duration of Amputation (y)	9.7 ± 8.7	7.4 ± 5.5	12.7 ± 11.2
Classification			
Right Transtibial	15	11	4
Left Transtibial	8	3	5
Right Transfemoral	2	1	1
Left Transfemoral	7	4	3
Bilateral Transtibial	3	0	3

Note: Values are (m ± sd). BMI is Body Mass Index.

**Table 2:** Means, standard deviations, and variable statistics for normal distribution.

	Balance Task				
	N	Mean	SD	Skewness	Kurtosis
EOS (%)	35	88.9	4.7	−2.58 *	0.34
EOD (cm)	35	41.5	26.3	5.91 *	8.64 *
EOV (cm/s)	35	1.38	0.87	5.90 *	8.62 *
ECS (%)	35	83.3	10.2	−5.29 *	6.13 *
ECD (cm)	35	86.7	65.4	4.21 *	2.78 *
ECV (cm/s)	35	2.81	2.07	4.60 *	4.00 *
	**Arm Crank Exercise**				
	**N**	**Mean**	**SD**	**Skewness**	**Kurtosis**
TTE (s)	35	422	158	1.46	−0.31
Power_peak_ (W)	35	59.1	20.6	1.44	−0.67
HR_peak_ (b/min)	29	143	30	−0.44	−0.62
VO_2peak_ (mL/kg/min)	34	15.7	7.6	2.13 *	0.21
VE_peak_ (L/min)	34	52.5	25.5	2.21 *	0.77
RER_peak_ (VCO_2_/VO_2_)	34	1.19	0.12	−0.97	−0.18
RPE_peak_	26	15.2	2.5	−0.19	0.50

* >1.96 represents violation of parametric assumption. Note: EOS is eyes open stability score, EOD is eyes open center of pressure displacement, EOV is eyes open center of pressure displacement velocity, ECS is eyes closed stability score, ECD is eyes closed center of pressure displacement, ECV is eyes closed center of pressure displacement velocity, TTE is time to exhaustion, Power_peak_ is peaks watts, HR_peak_ is peak heart rate, VO_2peak_ is peak oxygen consumption, VE_peak_ is peak ventilation, RER_peak_ is peak respiratory exchange ratio, and RPE_peak_ is peak rating of perceived exertion.

**Table 3:** Absolute values (mean ± sd) and mean ranks for balance and physiological variables between lowest and highest performers on arm crank exercise.

Balance Tasks					
	Lowest Performers (*n* = 12)	Highest Performers (*n* = 11)			
	Absolute Value (Mean Rank)	Absolute Value (Mean Rank)	*U*	Z-Value	*p*-Value
EOS	89.3 ± 4.9 (13.1)	88.3 ± 4.3 (10.7)	52.5	−0.84	0.402
EOD	16.4 ± 8.9 (14.7)	16.1 ± 11.9 (9.1)	34.0	−1.96	0.049 *
EOV	0.54 ± 0.29 (15.6)	0.53 ± 0.39 (9.2)	35.0	−1.91	0.056
ECS	82.2 ± 10.4 (10.1)	84.4 ± 10.0 (14.1)	43.5	−1.39	0.164
ECD	36.3 ± 25.8 (14.5)	31.8 ± 26.3 (9.3)	36.0	−1.84	0.065
ECV	1.2 ± 0.86 (14.7)	0.99 ± 0.78 (9.0)	33.0	−2.03	0.042 *
**Physiological Responses**					
TTE	303 ± 70 (6.5)	548 ± 12 (18.0)	0.00	−4.06	0.001 *
Power_peak_	43.5 ± 9.1 (6.5)	75.5 ± 16.1 (18.0)	0.00	−4.15	0.001 *
HR_peak_	136 ± 34 (7.6)	150 ± 23 (11.9)	21.0	−1.69	0.091
VO_2peak_	12.0 ± 5.2 (7.6)	19.8 ± 7.8 (16.2)	13.0	−3.09	0.002 *
VE_peak_	36.7 ± 12.7 (6.6)	70.1 ± 21.8 (17.4)	1.00	−3.89	0.001 *
RER_peak_	1.1 ± 0.1 (8.2)	1.2 ± 0.1 (15.4)	20.5	−2.07	0.009 *
RPE_peak_	15.3 ± 2.9 (7.9)	15.0 ± 2.0 (10.0)	27.0	−0.88	0.378

* Mean ranks are significantly different at the 0.05 level (2-tailed). Note: EOS is eyes open stability score (%), EOD is eyes open center of pressure displacement (cm), EOV is eyes open center of pressure displacement velocity (cm/s), ECS is eyes closed stability score (%), ECD is eyes closed center of pressure displacement (cm), ECV is eyes closed center of pressure displacement velocity (cm/s). TTE is time to exhaustion (s), Power_peak_ is peak power (W), HR_peak_ is peak heart rate (b/min), VO_2_peak is peak oxygen consumption (mL/kg/min), VE_peak_ is peak ventilation (L/min), RER_peak_ is peak respiratory exchange ratio (VCO_2_/VO_2_), and RPE_peak_ is peak rating of perceived exertion (Borg’s 6–20 scale).

## Data Availability

The data presented in this study are available on request from the corresponding author. The data are not publicly available due to a privacy agreement.
